# Artificial selection for odor-guided behavior in *Drosophila* reveals changes in food consumption

**DOI:** 10.1186/s12864-017-4233-1

**Published:** 2017-11-13

**Authors:** Elizabeth B. Brown, Cody Patterson, Rayanne Pancoast, Stephanie M. Rollmann

**Affiliations:** 1 Department of Biological Sciences, University of Cincinnati, Cincinnati, OH 45221-0006 USA; 2 Department of Biology, Xavier University, Cincinnati, OH 45207 USA

**Keywords:** *Drosophila*, Olfaction, Feeding, RNA sequencing, RNA interference

## Abstract

**Background:**

The olfactory system enables organisms to detect chemical cues in the environment and can signal the availability of food or the presence of a predator. Appropriate behavioral responses to these chemical cues are therefore important for organismal survival and can influence traits such as organismal life span and food consumption. However, understanding the genetic mechanisms underlying odor-guided behavior, correlated responses in other traits, and how these constrain or promote their evolution, remain an important challenge. Here, we performed artificial selection for attractive and aversive behavioral responses to four chemical compounds, two aromatics (4-ethylguaiacol and 4-methylphenol) and two esters (methyl hexanoate and ethyl acetate), for thirty generations.

**Results:**

Artificial selection for odor-guided behavior revealed symmetrical responses to selection for each of the four chemical compounds. We then investigated whether selection for odor-guided behavior resulted in correlated responses in life history traits and/or food consumption. We found changes in food consumption upon selection for behavioral responses to aromatics. In many cases, lines selected for increased attraction to aromatics showed an increase in food consumption. We then performed RNA sequencing of lines selected for responses to 4-ethylguaiacol to identify candidate genes associated with odor-guided behavior and its impact on food consumption. We identified 91 genes that were differentially expressed among lines, many of which were associated with metabolic processes. RNAi-mediated knockdown of select candidate genes further supports their role in odor-guided behavior and/or food consumption.

**Conclusions:**

This study identifies novel genes underlying variation in odor-guided behavior and further elucidates the genetic mechanisms underlying the interrelationship between olfaction and feeding.

**Electronic supplementary material:**

The online version of this article (10.1186/s12864-017-4233-1) contains supplementary material, which is available to authorized users.

## Background

Sensory systems enable organisms to interact with the environment. Whether avoiding a predator, seeking a mate, or searching for food, these behaviors are mediated through the sensory detection and subsequent processing of environmental cues. In the case of olfaction, aversive and attractive olfactory cues are used, for instance, by organisms to locate and evaluate food resources. Moreover, olfactory cues can also influence other traits, such as organismal life span and starvation resistance [1–5]. Given their importance to survival and reproduction, behavioral geneticists have long sought to understand the proximate mechanisms underlying olfactory behavior, its interrelationship with other traits, and the mechanisms governing or constraining its phenotypic evolution. Thus, remarkable progress has been made in uncovering the neural circuitry underlying the detection of chemical cues [6–8]. However, understanding how sensory input is processed to result in divergent behavioral responses (olfactory attraction or aversion) and the genetic mechanisms that underlie its association with traits such as feeding and life span remains an important challenge.

*Drosophila melanogaster* has emerged as a model system for investigating the genetic factors underlying the detection and discrimination of olfactory cues [6, 8]. Odorants are detected by odorant receptors expressed in olfactory sensory neurons (OSNs) located on either of two olfactory organs, the third segment of the antenna or the maxillary palp. Each OSN typically expresses a single odorant receptor (OR) type and projects its axons to a distinct glomerulus in the antennal lobe [9–13]. These odorant receptors comprise a family of sixty genes that together with the highly conserved co-receptor, *Orco*, are believed to form ligand-gated ion channels [10, 14–18]. First order OSNs form synaptic connections with second order projection neurons, which extend their axons to the mushroom body and lateral horn regions of the brain [19–22]. The resulting spatial and temporal representation of glomerular activity allows for the discrimination among the diverse odors present in the environment [10, 23, 24]. More recently, studies have also focused on the identification of genes associated with variation in olfactory behavior through the use of candidate gene association studies [25–29] as well as genome-wide association (GWA) mapping [30–32]. These analyses identified genes that form a pleiotropic network of interactions and are largely involved in nervous system development and function.

In addition, studies in *Drosophila* have focused on examining the interrelationship between olfaction, feeding, and life history traits, including longevity and starvation resistance [3, 4, 33, 34]. Changes in sensory perception, for example, through silencing of the co-receptor *Orco*, resulted in extended longevity and increased resistance to starvation. Altered metabolism was also observed, with sex-specific changes in triglyceride levels [3]. Additionally, the olfactory system is also associated with mediating feeding [35–38]. A starvation-dependent shift in OSN sensitivity via short neuropeptide F, a fly homolog of neuropeptide Y (NPY) [39], and insulin signaling regulates food search behavior [40]. Moreover, neurons expressing neuropeptide F (dNPF) respond to food odors, increasing in activity with increased food-odor attractiveness [41].

Here, we conduct artificial selection experiments to investigate the mechanisms underlying olfactory behavior and correlated responses with other traits. We independently selected for attractive and aversive behavioral responses to two aromatic compounds (4-ethylguaiacol and 4-methylphenol) and two esters (ethyl acetate and methyl hexanoate) for thirty generations. These odorants are a natural byproduct of yeast fermentation [42–45], a component of the feeding and breeding substrate of *Drosophila*. We then used these artificially selected lines to investigate potential relationships with other traits and found odor- and sex-specific differences in food consumption. We subsequently performed RNA sequencing (RNA-seq) experiments to identify changes in gene expression using lines selected for differences in behavioral responses to the odorant 4-ethylguaiacol and examined the role of a subset of these candidate genes in mediating odor-guided behavior and food consumption.

## Results

### Selection for odor-guided behavior

**Fig. 1:**
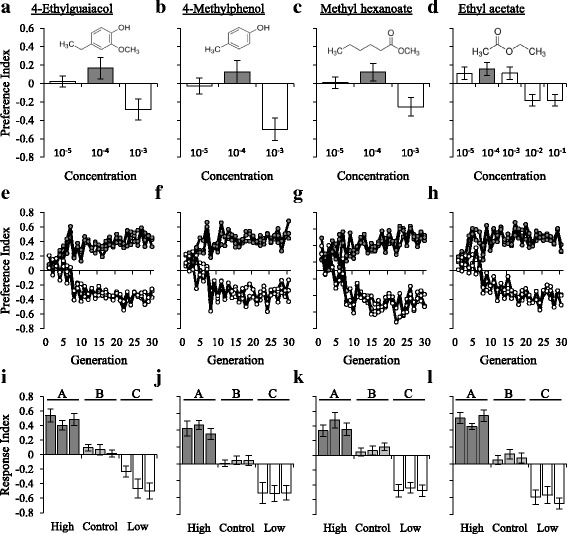
Odor-guided behavioral responses to four odorants. Row 1: Dose response curves of the base population to four odorants. Preference indexes for (**a**) 4-ethylguaiacol, (**b**) 4-methylphenol, (**c**) methyl hexanoate, and (**d**) ethyl acetate are shown. Gray bars indicate the concentration used in the artificial selection experiments. Data shown are means ± SE. *N* = 20. Row 2: Phenotypic response to artificial selection for odor-guided behavioral responses to (**e**) 4-ethylguaiacol, (**f**) 4-methylphenol, (**g**) methyl hexanoate, and (**h**) ethyl acetate. Mean preference index at each generation for the three replicate high selected lines (gray filled dots) and three low selected lines (white filled dots) are shown. *N* = 6. Row 3: Symmetrical response to selection for (**i**) 4-ethylguaiacol, (**j**) 4-methylphenol, (**k**) methyl hexanoate, and (**l**) ethyl acetate. Data shown are means ± SE. Letters indicate *P* < 0.05 using Tukey’s post hoc test. N = 20

To understand the genetic mechanisms underlying shifts in odor-guided behavior, we performed artificial selection experiments using the Flyland population, an outbred population derived from the *Drosophila* Genetic Reference Panel [46]. We measured its behavioral responses to four odorants, 4-ethylguaiacol (4EG), 4-methylphenol (4MP), methyl hexanoate (MH), and ethyl acetate (EA), at several different concentrations. We observed concentration-specific differences in behavioral responses to each odorant tested (Fig. 1a-d). Based on these results, we independently generated three replicate high and low lines selected for attractive or aversive behavioral responses to each odorant, as well as three replicate unselected (control) lines. After seven generations, mean high and low behavioral responses to all odorants significantly diverged relative to the controls, and plateaued at approximately generation ten (Fig. 1e-h, Additional file 1: Table S1). The response to selection was symmetrical, with responses of the controls intermediate between the high and low lines (Fig. 1i-l). No significant changes in locomotion were observed among the selection regimes (Additional file 2: Figure S1).

### Correlated responses to selection for odor-guided behavior

Changes in olfactory perception have been shown to affect life history traits, such as longevity and starvation resistance [3–5]. To test whether artificial selection for odor-guided behavior resulted in correlated responses in these traits, we measured longevity and starvation resistance across all four selection regimes. Overall no significant differences in life span were observed (Additional file 3: Figure S2; Additional file 1: Table S3). Measurements of starvation resistance, a trait often positively correlated with life span [47], also revealed no differences among lines in all four selection regimes (Additional file 4: Figure S3; Additional file 1: Table S3). One exception to these results was found for measurements of life span among the MH selected lines. Females selected for increased attraction to MH had a significant increase in life span relative to the control and low lines (Additional file 3: Figure S2).

**Fig. 2:**
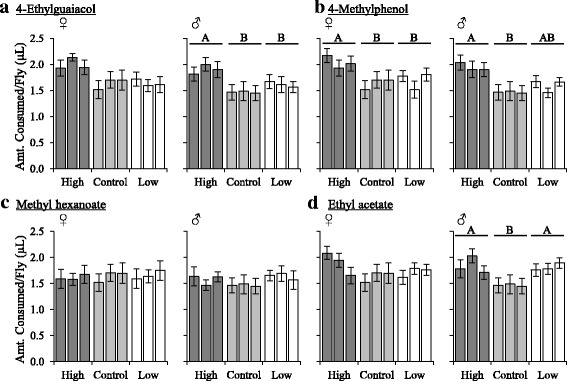
Food consumption measurements of lines selected for increased and decreased behavioral responses to (**a**) 4-ethylguaiacol, (**b**) 4-methylphenol, (**c**) methyl hexanoate, and (**d**) ethyl acetate using the CAFE assay. The total amount of yeast extract consumed was measured. Data shown are means ± SE for females (left) and males (right). Letters indicate *P* < 0.05 using Tukey’s post hoc test. *N* = 12

Alterations in chemosensory perception can also influence feeding behavior [35–38]. We therefore investigated whether changes in odor-guided behavior were associated with changes in food consumption using the CAFE assay [48]. When fed a standard liquid diet of sucrose and yeast extract, we observed significant sex-specific differences in food consumption among the 4EG and 4MP selected regimes in which high lines generally consumed significantly more than low and control lines (Fig. 2). In the case of lines selected for responses to 4EG, food consumption was significantly increased in males in the high selected lines, with the same trend apparent in females. For lines selected for responses to 4MP, food consumption was significantly increased in females in the high selected lines, with again a similar trend observed in males. This pattern of food consumption was not observed for lines selected for responses to the esters, MH and EA. In general, no significant changes in food consumption were observed among these lines, with the exception of males selected for responses to EA, in which both high and low lines showed increased consumption relative to the control.

Food consumption of the 4EG and 4MP selected lines were again tested, and differences in consumption observed using two additional diets. First, we replaced yeast extract with live *Brettanomyces* yeast. Regardless of sex, all 4EG and 4MP selected lines increased consumption overall. Moreover, consistent with previous results, we generally observed increased food consumption in high lines relative to low and control lines (Additional file 5: Figure S4a, b). Next, we fed flies a diet of sucrose only. We again observed significant differences in food consumption for both sexes, with high lines consuming more food than the low and control lines (Additional file 5: Figure S4c, d). Also, food consumption in the control lines remained below that of the low lines. Finally, we used a binary-choice assay to investigate whether sucrose supplemented with either 4EG or 4MP conferred differences in food preference [45]. Flies were given a choice between food with or without supplementation of odorant. We hypothesized we would observe an increase in preference for food supplemented with odorant in the high lines and reduced consumption in the low lines, given their attraction/aversion to each odorant, respectively. Interestingly, we found no significant differences in food preference, such that all selected lines equally, albeit mildly, preferred food supplemented with either 4EG or 4MP (Additional file 5: Figure S4e, f). Together, these results suggest that selection for behavioral responses to these aromatics results in correlated responses in feeding behavior with the high lines typically consuming more, irrespective of diet, but importantly that selection for increased attraction and aversion does not directly translate into corresponding changes in food consumption.

The changes in food consumption may result from changes in metabolic processes or from general differences in body mass [49]. To investigate whether selection for odor-guided behavior was also associated with changes in these traits, we measured dry mass in the 4EG and 4MP selected lines. For 4EG selected lines, no significant differences in body mass were observed (Additional file 6: Figure S5a). However, for the 4MP selected lines, the high selected lines weighed significantly more than the other lines (Additional file 6: Figure S5b). Measurements of metabolic processes (triglycerides, glucose, and glycogen) revealed no significant differences after adjustment for dry mass (Additional file 6: Figure S5c-h). One exception being males selected for behavioral responses to 4MP, in which the low lines had significantly lower glucose levels than the control lines, but neither of which were significantly different from the high lines. In short, for the 4EG and 4MP selection regimes, high lines consistently consumed more than low lines, however, these differences in food consumption cannot be clearly attributed to changes in the metabolic traits measured here.

### Transcriptional response to selection for odor-guided behavior

To examine the genetic mechanisms underlying differences in odor-guided behavior and the correlated responses on feeding, we conducted RNA-seq analyses on lines selected for divergent responses to 4EG. This selection regime was chosen for further analysis because differences were observed between the high and low selected lines for both odor-guided behavior and food consumption, independent of mass. Differential gene expression was examined in whole heads to include in the analysis genes associated with the peripheral olfactory organs, the brain, as well as feeding organs. In total, we obtained 780,640,808 100 bp reads from 18 cDNA libraries. Of 753,144,236 reads that passed quality filtering, 95.32% could be aligned to the *Drosophila* genome, and 98.64% of the aligned reads mapped to uniquely (Additional file 1: Table S4, Additional file 1: Table S5). Of the 17,471 annotated genes [50], 9238 had at least one read per million in at least half the samples. This set of genes was used for subsequent statistical analyses.

**Fig. 3:**
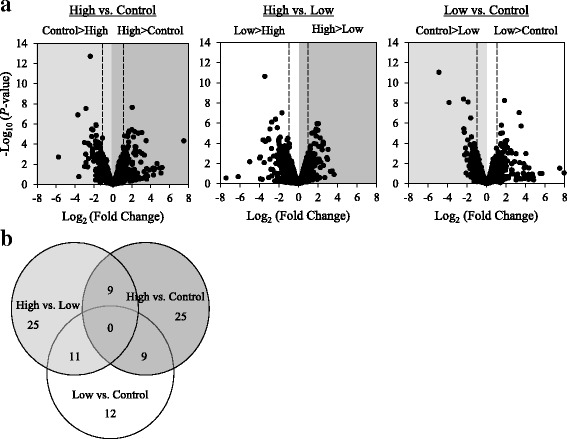
RNA-seq analyses of genes differentially expressed among lines selected for 4-ethylguaiacol. (**a**) Volcano-plot of RNA-seq results for all pairwise comparisons. For each comparison, genes to the left of the > symbol are upregulated, while genes to the right are downregulated. Vertical dashed lines represent a two-fold cutoff. (**b**) Venn diagram illustrates the differentially expressed genes that overlap between each comparison

Differential gene expression was evaluated between the different combinations of selection regimes, i.e., high vs. control, low vs. control, and high vs. low. A total of 43, 32, and 45 genes were significantly differentially expressed in the high vs. control, low vs. control, and high vs. low comparisons, respectively (Additional file 4: Fig. 3a; Additional file 1: Table S6–8). Eleven genes were shared between high vs. low and low vs. control comparisons, thereby highlighting genes that may contribute to aversive behavioral responses to 4EG. Nine genes were shared between high vs. control and low vs. control comparisons which may consist of genes that are responsible for generalized changes in behavior, regardless of hedonic value, as these genes were differentially expressed regardless of selection for high or low behavioral responses. Nine genes were also shared between the high vs. control and high vs. low comparisons and could give insights into the genes responsible for attraction to 4EG and its link with food consumption (Fig. 3b). Finally, we chose to focus on the high vs. low differentially expressed genes for subsequent analyses. We assessed whether there was overrepresentation of Gene Ontology (GO) terms. No significant overrepresentation for any GO terms were found (Additional file 7: Figure S6). However, of the 45 significantly differentially expressed genes, 13 were annotated for the GO term metabolic process and four for response to stimulus.

### Functional tests of candidate genes

**Fig. 4:**
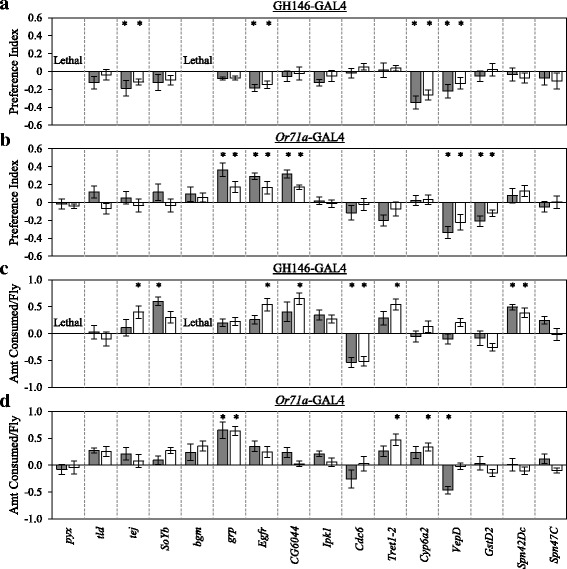
Functional tests of candidate genes and their effects on (**a**), (**b**) odor-guided behavioral responses to 4-ethylguaiacol and (**c**), (**d**) food consumption. For each trait, the effects of RNAi-mediated knockdown of 16 candidate genes in either projection neurons of the antennal lobe (GH146-GAL4) or in *Or71a*-expressing neurons (*Or71a*-GAL4) were examined in both females (gray bars) and males (white bars). Values are deviations from the control. Error bars indicate SE. *: *P* < 0.05. *N* = 10

To further assess the candidate genes significantly differentially expressed between high and low selected lines for their contribution to changes in odor-guided behavior and/or food consumption, we used RNA interference [51, 52] and the GAL4/UAS system [53] to knockdown gene expression. We selected a subset of 16 genes based on their statistical level of significance, their identification across treatments, and their GO classification. Since OSNs and projection neurons are two primary relay stations at which olfactory information is processed, we knocked down gene expression in OSNs expressing *Or71a*, an odorant receptor shown to be tuned to 4EG using the *Or71a*-GAL4 driver line, and in projection neurons of the antennal lobe using the GH146-GAL4 driver line [54]. We observed significant differences in odor-guided behavior for seven of the genes tested (Fig. 4a.b; Additional file 1: Table S9; *CG6044*, *Cyp6a2*, *Egfr*, *grp*, *GstD2*, *tej*, *VepD*), and differences in food consumption for 10 genes (Fig. 4c, d; Additional file 1: Table S9; *Cdc6*, *CG6044*, *Cyp6a2*, *Egfr*, *grp*, *SoYb*, *Spn42Dc*, *tej*, *Tret1*–2, *VepD*). RNAi targeting of six of these genes resulted in significant differences in both odor-guided behavior and food consumption (*CG6044*, *Cyp6a2*, *Egfr*, *grp*, *tej*, *VepD*). The effects on behavior depended on sex, and the gene and/or neuronal population targeted. For example, expression of *grp*-RNAi in *Or71a*-expressing OSNs resulted in increased behavioral responses to 4EG as well as an increase in food consumption. However, a similar positive response was not always observed, as was the case with RNAi-mediated knockdown of *Egfr* and *tej* in projection neurons.

## Discussion

### Selection for odor-guided behavior

Artificial selection is a powerful method that allows us to examine divergence in odor-guided behavioral responses and how selection for attraction and aversion may result in correlated responses with other traits. Using this approach, selection lines were generated for odor-guided behavioral responses to four odorants, two aromatics and two esters. The response to selection was symmetric, such that both lines selected for attractive and aversive behavioral responses significantly differed relative to control. We then used these artificially selected lines to investigate correlated responses with odor-guided behavior and observed changes in food consumption associated with selection for behavioral responses to aromatics.

These aromatic compounds are detected by receptors expressed in olfactory sensory neurons of the maxillary palp. Detection of olfactory cues via the maxillary palp has been posited to mediate taste enhancement [55], with OSNs from the palp projecting to both the antennal lobe as well as the subesophageal ganglion, the primary taste center of the brain [56]. More specifically, these odorants (4EG and 4MP) bind to odorant receptor 71a (*Or71a*), which is expressed in palp OSNs [45, 57, 58]. Odor-evoked responses of *Or71a*-expressing neurons are of particular interest because previous studies suggest that detection of ethylphenols, including 4EG, by these neurons serves as a proxy for the detection of dietary antioxidants. Moreover, thermogenetic activation of these neurons via expression of dTRPA1 resulted in changes in feeding [45]. Thus, together with previous findings, our study sets the stage for future dissection of how changes in the maxillary palp may affect these behaviors.

In this study, the bidirectional response to selection for behavioral responses to aromatics resulted in an asymmetrical effect on food consumption. Significant increases in food consumption were typically observed in the high lines, relative to the low and control lines, irrespective of diet. Furthermore, we hypothesized that olfactory aversion would be associated with reduced food consumption, particularly when food was supplemented with the ‘aversive’ odorant. In point of fact, this was not the case. Previous studies have suggested that the context in which an odor is detected can be an important modulator of behavior [59]. For example, CO_2_, a chemical compound emitted from stressed flies, results in robust aversive behavioral responses [60]. However, when this odorant is paired with food odors, the aversive response is suppressed [61, 62]. Such context dependent behavior is modulated at multiple levels of the olfactory circuitry, from the periphery to the brain. This study suggests that the mechanisms underlying olfactory behavior and food consumption are partially independent and again suggests that the behaviors may be context dependent.

Finally, correlated changes in food consumption may result from changes in metabolism or body mass [49]. Yet, for both selection regimes we observed little to no concomitant shift in traits associated with metabolism. It is possible that correlated changes in food consumption are indeed associated with changes in the metabolic traits measured here, but not at levels sufficient for detection. Alternatively, potential differences in other metabolic traits not measured in this study, such as metabolic rate, could underlie the observed differences in food consumption [63]. In the case of dry mass, a difference among lines was only observed in the 4MP selection regime despite changes in both food consumption and odor-guided behavioral responses being observed in both 4EG and 4MP selection regimes. This suggests that the mechanisms underlying variation in responses to these odorants may at least partially differ.

### Transcriptional response to selection for odor-guided behavior

To further understand the mechanisms underlying the divergence in odor-guided behavior and its association with food consumption, we conducted an RNA-seq experiment using lines selected for attractive and aversive behavioral responses to 4EG. We hypothesized that we would identify odorant receptor and odorant binding protein genes, as these have been previously associated with variation in odor-guided behavior [25, 26, 28, 29, 64], most notably *Or71a*, because 4EG is a ligand of this receptor [45]. Contrary to our expectations, however, we did not observe differences in expression of *Or71a*, nor in any other odorant receptor or odorant binding protein genes. Interestingly, however, we did identify genes (*Cyp6a8*, *Cyp6a20*, *Cyp6a2*, *Cyp4d1*, *GstD2*) belonging to the cytochrome P450 and glutathione S-transferase families, which have been implicated in odor signal termination [65]. The rapid degradation of odors, and subsequent termination of the odor signal, enables an organism to appropriately behaviorally respond to volatile changes in the environment. Therefore, changes in the expression of these genes may alter odor perception. Moreover, of the 91 differentially expressed genes identified in our study, 19 were also identified in genome-wide association analyses (GWA) on odor-guided behavior (*ACXD*, *bgm*, *Cdc6*, CG5773, CG8785, *CG9616*, *CG10064*, *CG10205*, *CG12374*, *CG14608*, *CG31809*, *CG34273*, *CG43448*, *Cpr66D*, *Cyp6a8*, *Egfr*, *Hel89B*, *luna*, *pwn*) [30–32]. These overlapping candidate genes were identified despite using different odorants with varying valence, thereby suggesting that they indeed may function in odor-guided behavior regardless of odor intensity or hedonic value. Finally, our analyses revealed novel genes not previously implicated in mediating odor-guided behavior and afford new insights into the genetic mechanisms underlying these behavioral responses.

Given the observed differences in food consumption, we also hypothesized that we would identify genes involved in the insulin signaling and dNPF pathways, as both pathways are known to regulate multiple aspects of feeding behavior [66–69]. However, no differentially expressed genes were associated directly with these pathways. This can possibly be attributed to their high degree of conservation [39, 70], given that many of these genes are selectively constrained [71, 72]. However, our analyses did reveal differential expression of genes involved in metabolism (*bgm*, *CG2469*, *CG10116*, *CG10962*, *CG33958*, *Cyp4d1*, *GstD2*, *HIP*, *Ipk1*, *phr*, *SP1029*, *tej*, *tld*). Our analyses also revealed two genes identified in GWA analyses to be associated with variation in food consumption (*CG43448*, *Egfr*) [73], and three that overlap with the genomic response to selection for increased feeding (*CG14696*, *Egfr*, *tld*) [74]. The identification of these candidate genes in our study raises the possibility that they function at the interface between olfaction and feeding.

### Functional assessment of candidate genes

We functionally assessed the effects of 16 candidate genes in *Or71a*-expressing neurons and projection neurons of the olfactory system. RNAi mediated targeting of eleven candidate genes resulted in changes in olfactory and/or food consumption. The remaining five genes did not significantly influence behavior. We cannot exclude the possibility, however, that these genes influence these traits through other mechanisms not tested in this study. Of those that did influence behavior in our study, several genes were of note. RNAi-targeting of Epidermal growth factor receptor (*Egfr*) resulted in a significant decrease in attraction when it was knocked down in projection neurons, but an opposite effect in *Or71a*-expressing neurons. This gene plays an extensive role in development, such as in cell fate specification [75–78]. Additionally, it has been implicated in GWA studies of *Drosophila* olfactory and feeding behaviors [32, 73] as well in mammalian obesity [79, 80]. Moreover, RNAi-mediated knockdown of Trehalose transporter 1–2 (*Tret1–2*) resulted in increased food consumption in males. A structurally similar gene, *Tret1–1*, regulates the release of trehalose, the primary sugar in insect hemolymph [81]. Although, *Tret1–2* does not function in trehalose transport [81], our results suggest a role for *Tret1–2* in mediating food consumption.

Finally, perhaps one of the most intriguing genes was grapes (*grp*). This gene had effects on both odor-guided behavior and food consumption. The directionality of *grp* expression is similar in both our RNA-seq analyses and RNAi experiments. High selected lines had significantly lower *grp* expression than low selected lines, and knockdown of *grp* expression in *Or71a*-expressing neurons recapitulated this pattern with a significant increase in attraction to 4EG and significantly higher amounts of food consumed relative to the control. This gene is expressed in the antenna [82], involved in sensory organ development [83], and has been previously identified in a GWA analysis on odor-guided behavior [32]. Moreover, *grp* belongs to the calcium/calmodulin-dependent protein kinase gene family [84–86]. Another member of this gene family, Ca^2+^/calmodulin kinase II (*CaMKII*), is not differentially expressed in this study, but is well known for its role in olfaction. This gene regulates the termination of olfactory signaling by olfactory adenylyl cyclase [87] and has been implicated in olfactory memory formation [88]. Members of the calcium/calmodulin-dependent protein kinase family have also been implicated in the regulation of metabolism. In rats, administration of NPY to the hypothalamus, a region of the brain controlling appetite [89], increases CaMKII activity [90]. Two additional members of this family, CaMKI and CaMKIV, stimulate insulin biosynthesis in pancreatic β-cells in response to glucose stimulation [91]. The involvement of this gene family in olfaction as well as the metabolic control of feeding sets the stage for future investigations into the molecular mechanisms by which *grp* affects olfactory perception and food consumption.

## Conclusions

Artificial selection for attractive and aversive behavioral responses to four odorants revealed symmetrical responses to selection, such that the selection lines differed in their behavioral responses relative to the controls. Measurements of food consumption were associated with selection for behavioral responses to 4EG and 4MP. Differential expression analyses revealed genes involved in metabolism in lines selected for behavioral responses to 4EG. RNAi mediated knockdown of gene expression of several of these candidate genes revealed their role within specific neuronal populations of the olfactory system and sets the stage for future work on the functional mechanisms by which these genes affect both odor-guided behavior and food consumption. This work provides novel insights into the genetic architecture underlying olfactory behavior and its association with feeding behavior.

## Methods

### *Drosophila* maintenance and husbandry

Flies were reared on standard cornmeal/agar/molasses media at 25 °C under a 12 h light–dark cycle. Flyland was kindly provided by Dr. Trudy F. Mackay [46]. Transgenic RNAi lines obtained from the *Drosophila* Transgenic RNAi Project (Harvard Medical School) include: *pyx* (31297), *tld* (51507), *tej* (36879), *SoYb* (36881), *bgm* (55918), *grp* (36685), *Egfr* (36773), *CG6044* (28610), *Ipk1* (35250), *Cdc6* (55734), as well as their co-isogenic controls attP2 (36303) and attP40 (36304) [51, 52]. RNAi lines were also obtained from the Vienna *Drosophila* Resource Center [92]. These lines include: *Tret1*–2 (v40980), *Cyp6a2* (v48849), *VepD* (v103259), *GstD2* (v109123), *Spn42Dc* (v13263), *Spn47C* (v100328) as well as the control lines *w* ^*1118*^ (v60000) and *y,w[1118];P{attP,y[+],w[3`]* (v60100). Each RNAi line was crossed to the following drivers: GH146-GAL4 (gift from F. Hamada, University of Cincinnati) and *Or71a*-GAL4 (23122). Controls for genetic background were generated by crossing GAL4 driver lines to the appropriate host strain used to generate the RNAi line.

### T-maze assay

Behavioral assays were conducted as previously described [93], with minor modification. Briefly, thirty flies were placed into the center of a T-maze apparatus and allowed to acclimate for one minute. Flies were then given a choice between the two arms of the maze, one arm containing the diluted odorant and the other containing the paraffin oil vehicle. Both arms had airflows of 500 mL/min. After one minute, the number of flies on each side were counted. The preference index (PI) was calculated using the formula: PI = (O – N) / (O + N), where O is the total number of flies on the odor side, N is the total number of flies on the side not containing odor. A positive PI is indicative of attraction to the odor (with a maximal response of +1), whereas a negative PI indicates repulsion (with a maximal response of −1). All assays were conducted in the morning in the dark at 25 °C and 70% humidity. All flies were aged 3–7 days post-eclosion and were starved overnight on 1% agar (MoorAgar Inc.; Rocklin, CA). Each line and sex were tested separately and the arm from which odor was emitted was randomized each day of testing. RNA interference experiments were conducted using the same protocol as above.

### Artificial selection

Artificial selection experiments were conducted for behavioral responses to four odorants: 4-ethylguaiacol (Sigma-Aldrich; St. Louis, MO), 4-methylphenol (Sigma-Aldrich), methyl hexanoate (Sigma-Aldrich), and ethyl acetate (Sigma-Aldrich), using Flyland as the base population [45]. All behavioral assays were performed at 0.01%. To commence selection, we measured behavioral responses of virgin females and males to each odorant. Upon completion of each assay, flies were collected from the odor- and non-odor sides of the T-maze. Flies collected from the odor side were used to establish the high lines, while files from the non-odor side were used to establish the low lines. Behavioral tests were performed until a minimum of 25 females and 25 males for each of three replicate high and low responding lines were obtained for each odorant. To establish the control lines, assays were conducted in which there was no odor present in the T-maze. Flies were then collected from one side of the T-maze, selected at random. This selection regime was repeated each generation for 30 generations. Symmetrical responses to selection were assessed at generation 18.

### Locomotion

Locomotor reactivity was measured as described previously [94]. Briefly, single flies were placed into vials containing standard food media and acclimated overnight. Locomotion was quantified by recording the amount of time a single fly was active over a 45 s time period immediately following a mechanical disturbance. For each line and sex, 10 replicate measurements were taken at generation 18.

### Correlated responses to selection

Food consumption was measured using the CAFE assay [48, 95]. Briefly, each chamber contained a calibrated glass micropipette (VWR, Radnor, PA) filled with 5 μl of liquid medium that was inserted through a foam plug and held in place with a pipette tip. At the bottom of each chamber, 1% agar was used as a water source. Flies were habituated to the chambers for 24 h with ad libitum food prior to testing. Food consumption was measured for 24 h. For feeding preference experiments, feeding preference was calculated using the formula: (O – C) / (O + C), where O is the amount of food consumed with supplementation of odorant and C is the amount of food consumed without odorant [45]. Identical chambers without flies were maintained to assess evaporation and the total amount consumed was adjusted accordingly. Flies tested were 2–4 days post-eclosion. Unless otherwise specified, the liquid food was 5% sucrose (Sigma-Aldrich), 5% yeast extract (Fisher Scientific, Hampton, NH) or live *Brettanomyces* yeast (William’s Brewing, San Leandro, CA), and 0.001% FD&C blue dye (Spectra Colors Corp., Kearny, NJ). For each line and sex, 12 replicates housing five flies each were tested.

For dry mass, longevity, and starvation resistance measurements, flies were reared as larvae at constant density of 50 larvae per vial. To measure dry mass, flies were separated by sex and placed on fresh media for 24 h after which they were dried for 24 h at 70 °C. For each line and sex, 10 replicate measurements were taken. Longevity was measured as previously described [96]. Briefly, 10 flies were placed into a vial and then scored every 24 h. Flies were transferred to fresh media every 2–3 days, during which dead flies were removed. For each line and sex, eight replicate vials were measured. For starvation resistance, flies were starved on 1% agar and survival measured every 8 h until death. For each line and sex, eight replicate vials of 10 flies each were measured. All experiments were performed on mated flies. Longevity and starvation resistance were measured at generation 18 and dry mass at 26.

Triglyceride (TAG), glucose, and glycogen levels were calculated as previously described [97]. For each line and sex, three replicate measurements consisting of five adult flies were taken at generation 28. Each sample was homogenized in 100 μl PBS, heat treated at 70 °C for 10 min, then flash frozen. Samples were subsequently thawed and assayed for glucose, glycogen and TAG content. Glucose and glycogen were quantified using the Glucose assay kit (Sigma-Aldrich). Glucose was measured directly from the homogenate, whereas glycogen was first digested to glucose during a 60 min incubation at 37 °C with 15 μl amyloglucosidase solution at 1.5 U ml^−1^ (Sigma-Aldrich), from which the previous glucose measurements were then subtracted. TAG levels were quantified by measuring glycerol content before and after digestion with Triglyceride Reagent (Sigma-Aldrich). TAG content was determined as the difference in glycerol content between the TAG-digested and TAG-undigested measurements.

### Statistical analysis

A Shapiro-Wilk test was performed for each experiment to assess normality prior to subsequent statistical analysis (data not shown). For measurements of olfactory behavior, food consumption, and nutrient stores, we performed a nested mixed model analysis of variance (ANOVA) that accounted for the number of replicate lines within each selection regime (high, low and control): Y = μ + Selection + Line (Selection) + Sex +Selection x Sex + Line (Selection) x Sex + ε. Where Selection is the fixed effect of selection treatment (high, control, or low behavioral responses), Line is the random effect of replicate within each selection regime, Sex is the fixed effect of sex, and ε indicates error. If no significant difference between sexes was observed, the data were pooled. *Post-hoc* analyses were conducted using Tukey’s HSD test. For starvation resistance and longevity measurements, log-rank tests were performed for survivorship analyses. For RNAi experiments, significant differences between the knockdown and its corresponding isogenic control were assessed using Dunnett’s tests for each sex. All data was analyzed using JMP 12.0 software (SAS Institute Inc., Cary, NC).

### RNA isolation and sequencing

Whole heads from 100 female adult flies aged 3–7 days post-eclosion were hand dissected in the morning. Two independent biological replicates were collected for each of the three high, low, and control lines. Total RNA was isolated using an RNeasy Mini Kit (Qiagen, Valencia, CA, 74,104). Total RNA was provided to the Weill Cornell Medical College Genomics Resources Core Facility for subsequent RNA sequencing using standard protocols, during which cDNA libraries were generated from each sample, and then sequenced using Illumina HiSeq4000 to generate 100 bp reads.

### RNA-seq processing and analysis

Adapters were removed from raw sequence reads using the program Trim Galore! (http://www.bioinformatics.babraham.ac.uk/projects/trim_galore), modifying the default parameters to allow a maximum error rate of zero. From there, the Cutadapt program was used to trim low quality sequences with Phred scores below 20 as well as remove reads shorter than 30 bp from the analysis [98]. The remaining RNA-seq reads were then aligned to the *Drosophila melanogaster* reference genome (version 6.10) [50] using STAR [99]. Differential expression between selection regimes was assessed using the Bioconductor EdgeR package [100]. Raw read counts were filtered to keep only genes that contain at least one read per million in at least half the samples. The data were then normalized for library size using the *calcNormFactors* function. To identify the differentially expressed genes between high, low, and control selected lines, three comparisons were performed: (a) high vs low, (b) high vs control, (c) low vs control. For each comparison, the three replicate lines composing each high, low, and control treatments were pooled. To account for multiple testing, we applied a FDR of 0.10. The program Panther was used to assess whether there was overrepresentation of Gene Ontology (GO) terms [101, 102].

## Additional files

Additional file 1: Table S1. Analyses of variance on odor-guided behavioral responses to artificial selection for each odorant at a given generation. **Table S2.** Analyses of variance on each trait measured. Traits include locomotor reactivity, longevity, starvation resistance, measurements of food consumption, dry mass, triglyceride levels, glucose, and glycogen. **Table S3.** Log-rank tests on longevity and starvation resistance measurements. **Table S4.** Summary of RNA-Seq datasets for control and 4-ethylguaiacol selected lines. Total reads are the number of reads that have passed quality filtering for each sample. Aligned reads and percent aligned are the number and percent of total reads that could be aligned to the reference genome. Uniquely mapped and percentage mapped reads are the number and percentage of reads that mapped uniquely. **Table S5.** RNA-seq results for control and 4-ethylguaiacol selected lines. For each gene, its FlyBase ID and the total number of raw reads in each sample is provided. **Table S6.** Differentially expressed genes between control and high lines selected for behavioral responses to 4-ethylguaiacol. For each gene, the log2 fold change (FC) and the log2 counts per million (CPM) are listed. Also listed is the corresponding likelihood ratio (LR), its *P*-value (*P*), and false discovery rate (FDR). **Table S7.** Differentially expressed genes between control and low lines selected for behavioral responses to 4-ethylguaiacol. For each gene, the log2 fold change (FC) and the log2 counts per million (CPM) are listed. Also listed is the corresponding likelihood ratio (LR), its *P*-value (*P*), and false discovery rate (FDR). **Table S8.** Differentially expressed genes between high and low lines selected for behavioral responses to 4-ethylguaiacol. For each gene, the log2 fold change (FC) and the log2 counts per million (CPM) are listed. Also listed is the corresponding likelihood ratio (LR), its *P*-value (*P*), and false discovery rate (FDR). **Table S9.** Dunnett’s test on odor-guided behavior and food consumption measurements for genes silenced in distinct neuronal subpopulations using RNA interference. For both odor-guided behavior and food consumption, the GAL4 driver lines tested, gene names, and *P*-value (*P*) are listed. (XLSX 4068 kb)

Additional file 2: Figure S1. Locomotor reactivity of (**a**) 4-ethylguaiacol, (**b**) 4-methylphenol, (**c**) methyl hexanoate, and (**d**) ethyl acetate selected lines. Data shown are means ± SE. *N* = 20. (PDF 16 kb)

Additional file 3: Figure S2. Longevity of lines selected for (**a, b**) 4-ethylguaiacol, (**c, d**) 4-methylphenol, (**e, f**) methyl hexanoate, and (**g, h**) ethyl acetate. For each line and sex, survivorship curves (panels 1 and 2) and median survivorship (panels 3 and 4) are shown. Data shown are median ± SE for females and males (left and right columns, respectively). *N* = 70. Letters indicate *P* < 0.05 using Tukey’s post hoc test. (PDF 126 kb)

Additional file 4: Figure S3. Starvation resistance of lines selected for (**a, b**) 4-ethylguaiacol, (**c, d**) 4-methylphenol, (**e, f**) methyl hexanoate, and (**g, h**) ethyl acetate. For each line and sex, survivorship curves (panels 1 and 2) and median survivorship (panels 3 and 4) are shown. Data shown are median ± SE for females and males (left and right columns, respectively). N = 70. (PDF 111 kb)

Additional file 5: Figure S4. Feeding measurements of lines selected for increased and decreased behavioral responses to 4-ethylguaiacol and 4-methylphenol using the CAFE assay. Row 1: Food consumption measurements of live *Brettanomyces* yeast for lines selected for (**a**) 4-ethylguaiacol and (**b**) 4-methylphenol. *N* = 12. Row 2: Food consumption measurements of sucrose for lines selected for (**c**) 4-ethylguaiacol and (**d**) 4-methylphenol. N = 12. Row 3: Binary preference assay for food with or without supplementation of either (**e**) 4-ethylguaiacol or (**f**) 4-methylphenol. Positive values indicate preference for food supplemented with odor. N = 12. Data shown are means ± SE for females (left) and males (right). Letters indicate *P* < 0.05 using Tukey’s post hoc test. (PDF 104 kb)

Additional file 6: Figure S5. Dry mass and measurements of metabolism. (**a, b**) Dry mass (*N* = 10), (**c, d**) adjusted triglyceride levels (*N* = 3), (**e, f**) adjusted glucose (N = 3), and (**g, h**) adjusted glycogen measurements (N = 3) for lines selected for increased and decreased behavioral responses to 4-ethylguaiacol and 4-methylphenol. Data shown are means ± SE for females and males (left and right columns, respectively). Letters indicate *P* < 0.05 using Tukey’s post hoc test. (PDF 207 kb)

Additional file 7: Figure S6. Categorization of differentially expressed genes among lines selected for differences in behavioral responses to 4-ethylguaiacol into (**a**) biological process, (**b**) molecular function, and (**c**) cellular component gene ontology terms. (PDF 18 kb)

## Abbreviations

χ^2^: Chi-square value
4EG: 4-ethylguaiacol
4MP: 4-methylphenol
CPM: Counts per million
Df: Degrees of freedom
EA: Ethyl acetate
*F*: *F* ratio
FC: Fold count
FDR: False discovery rate
GO: Gene ontology
GWA: Genome-wide association
LR: Likelihood ratio
MH: Methyl hexanoate
OSN: Olfactory sensory neuron
*P*: *P*-value
S.S.: Sum of squares
TAG: Triglyceride
