# Supplementary figures and images for: Artificial selection for odor-guided behavior in Drosophila reveals changes in food consumption
Source: BMC Genomics. 2017 Nov 13;18:867. doi: 10.1186/s12864-017-4233-1 (PMC5683340; doi:10.1186/s12864-017-4233-1)

**a** 4-Ethylguaiaicol

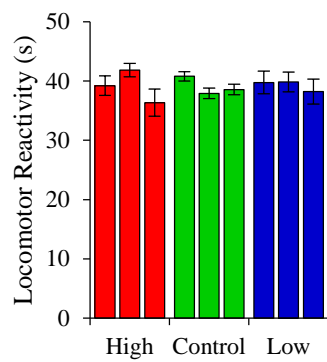

**b** 4-Methylphenol

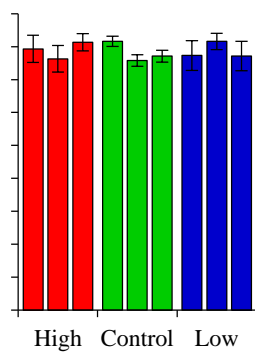

**c** Methyl hexanoate

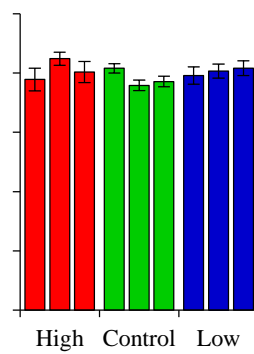

**d** Ethyl acetate

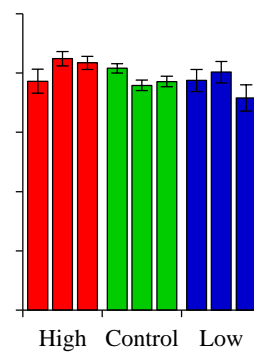

Supplement: Supplementary file 2 — Locomotor reactivity of (a) 4-ethylguaiacol, (b) 4-methylphenol, (c) methyl hexanoate, and (d) ethyl acetate selected lines. Data shown are means ± SE. N = 20. (PDF 16 kb) [file 12864_2017_4233_MOESM2_ESM.pdf]

**a** 4-Ethylguaiacol

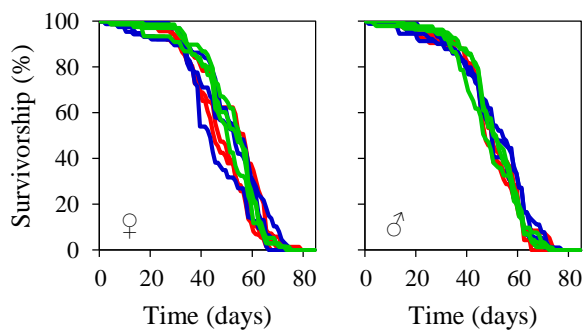

**b**

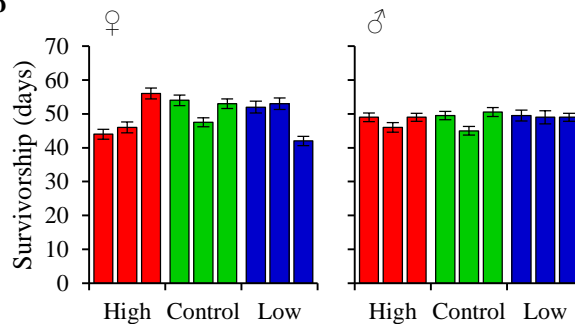

**c** 4-Methylphenol

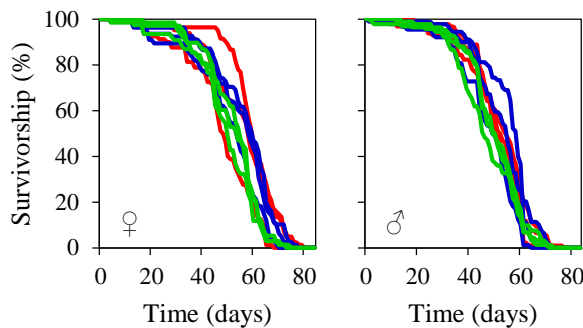

**d**

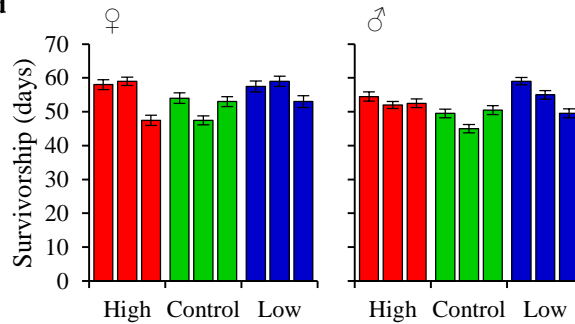

**e** Methyl hexanoate

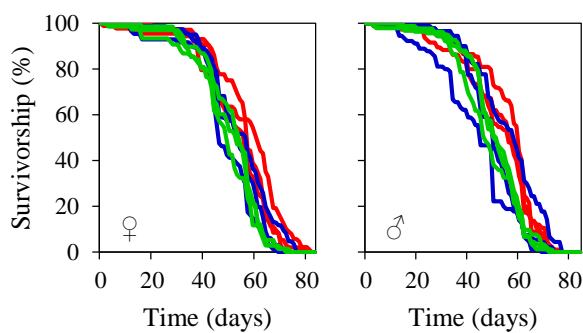

**f**

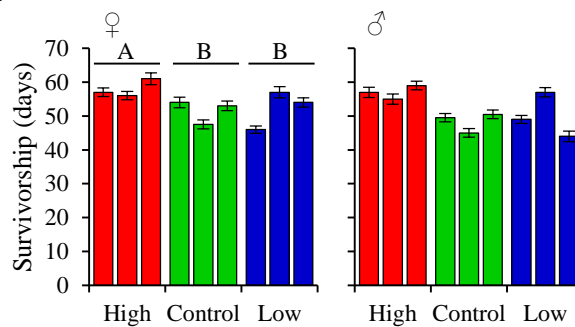

**g** Ethyl acetate

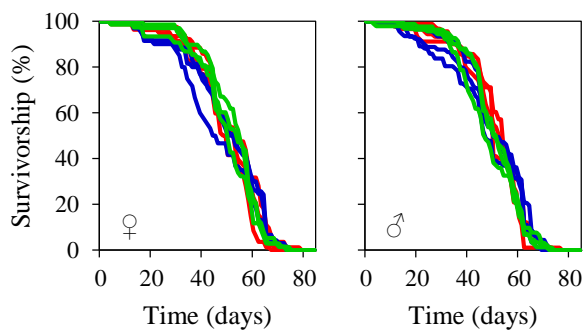

**h**

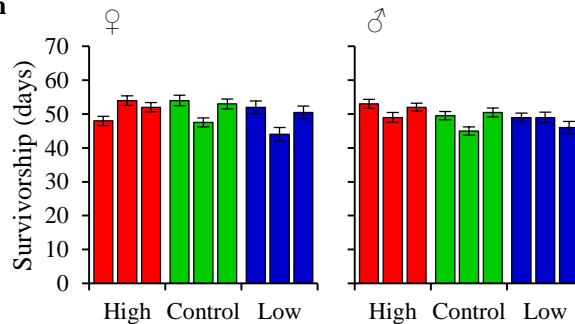

Supplement: Supplementary file 3 — Longevity of lines selected for (a, b) 4-ethylguaiacol, (c, d) 4-methylphenol, (e, f) methyl hexanoate, and (g, h) ethyl acetate. For each line and sex, survivorship curves (panels 1 and 2) and median survivorship (panels 3 and 4) are shown. Data shown are median ± SE for females and males (left and right columns, respectively). N = 70. Letters indicate P < 0.05 using Tukey’s post hoc test. (PDF 126 kb) [file 12864_2017_4233_MOESM3_ESM.pdf]

**a** 4-Ethylguaiacol

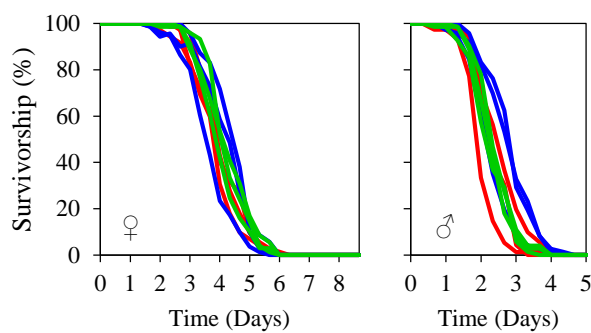

**b**

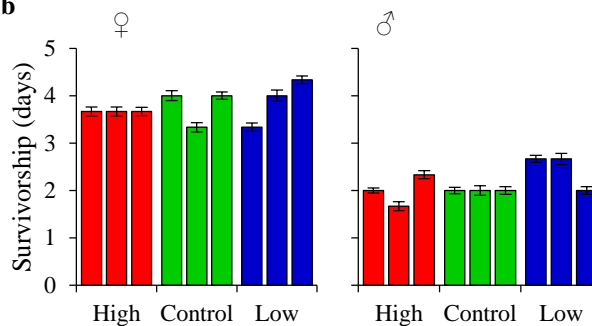

**c** 4-Methylphenol

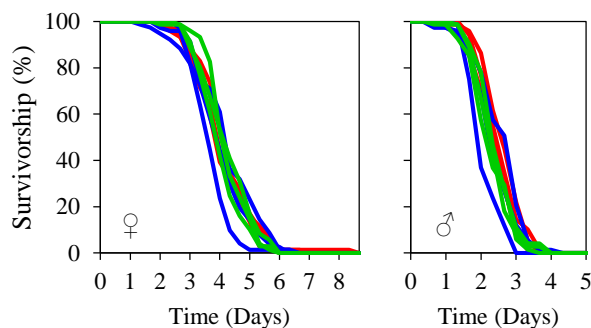

**d**

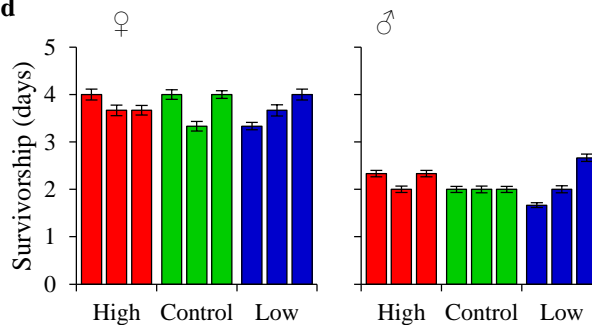

**e** Methyl hexanoate

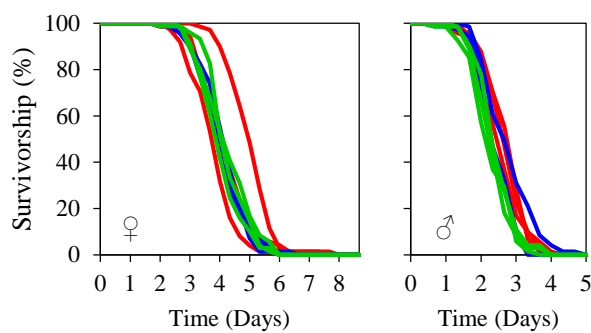

**f**

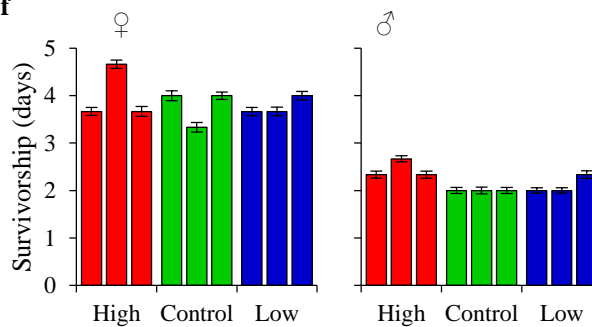

**g** Ethyl acetate

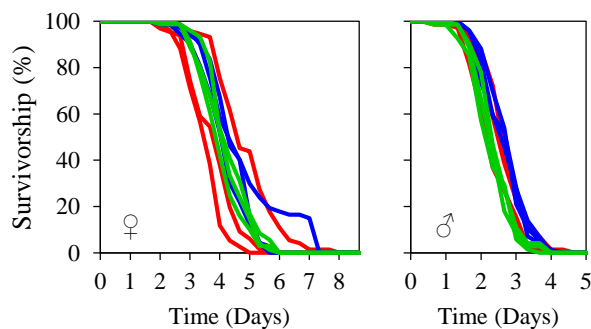

**h**

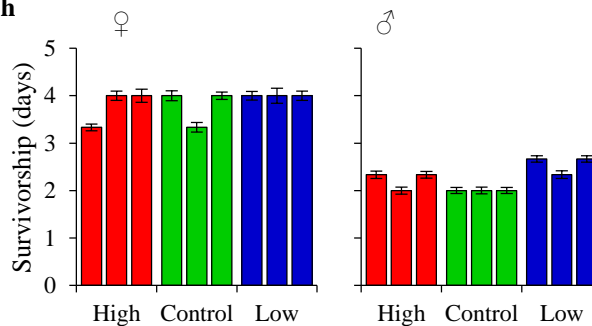

Supplement: Supplementary file 4 — Starvation resistance of lines selected for (a, b) 4-ethylguaiacol, (c, d) 4-methylphenol, (e, f) methyl hexanoate, and (g, h) ethyl acetate. For each line and sex, survivorship curves (panels 1 and 2) and median survivorship (panels 3 and 4) are shown. Data shown are median ± SE for females and males (left and right columns, respectively). N = 70. (PDF 111 kb) [file 12864_2017_4233_MOESM4_ESM.pdf]

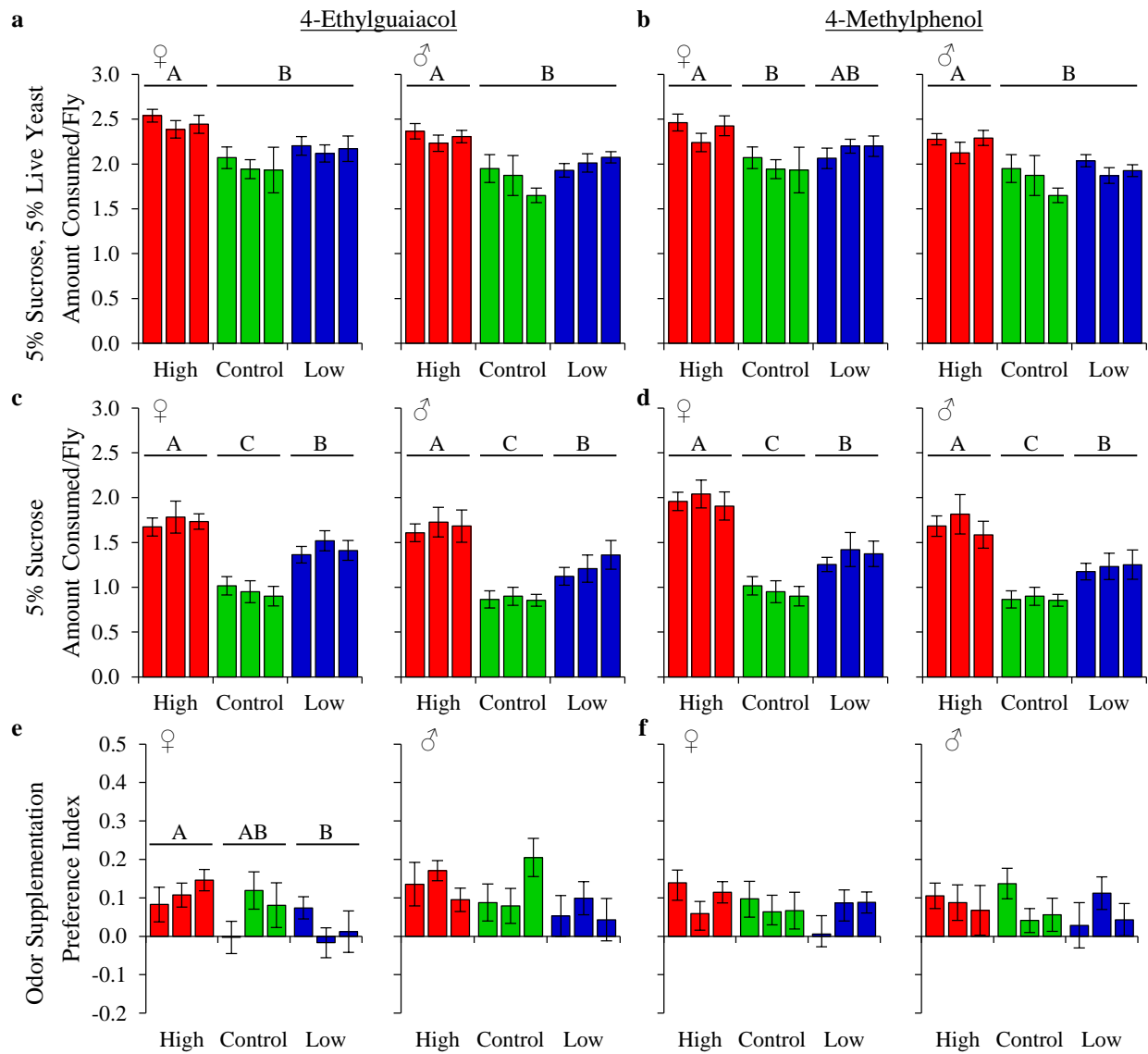

Supplement: Supplementary file 5 — Feeding measurements of lines selected for increased and decreased behavioral responses to 4-ethylguaiacol and 4-methylphenol using the CAFE assay. Row 1: Food consumption measurements of live Brettanomyces yeast for lines selected for (a) 4-ethylguaiacol and (b) 4-methylphenol. N = 12. Row 2: Food consumption measurements of sucrose for lines selected for (c) 4-ethylguaiacol and (d) 4-methylphenol. N = 12. Row 3: Binary preference assay for food with or without supplementation of either (e) 4-ethylguaiacol or (f) 4-methylphenol. Positive values indicate preference for food supplemented with odor. N = 12. Data shown are means ± SE for females (left) and males (right). Letters indicate P < 0.05 using Tukey’s post hoc test. (PDF 104 kb) [file 12864_2017_4233_MOESM5_ESM.pdf]

**a** 4-Ethylguaiacol

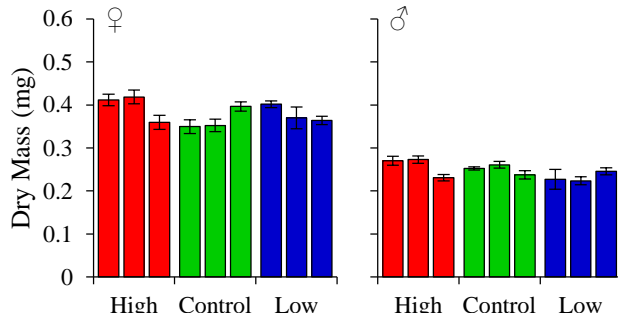

**b** 4-Methylphenol

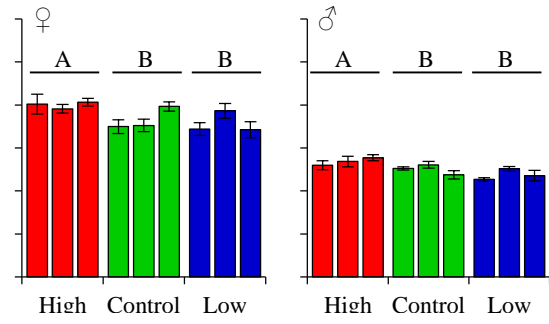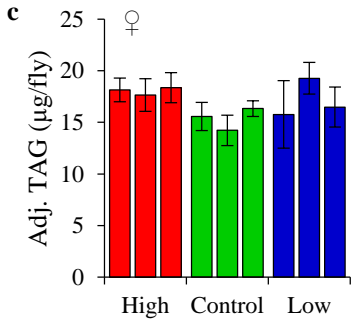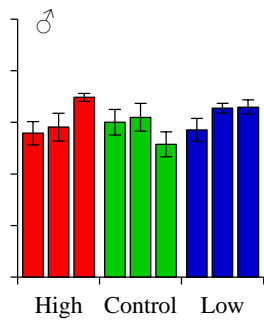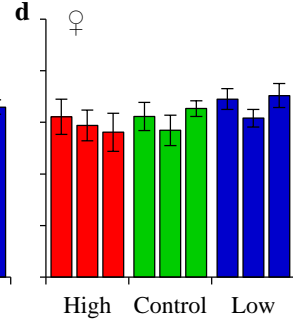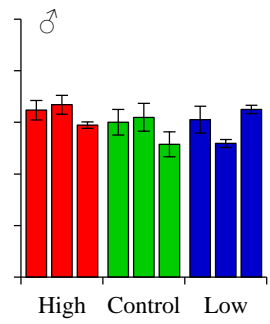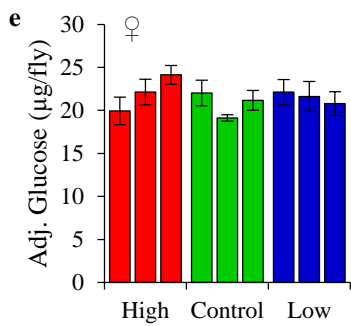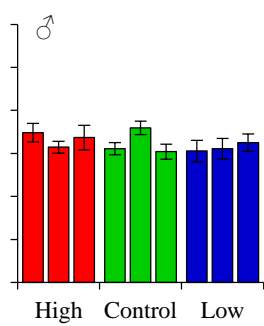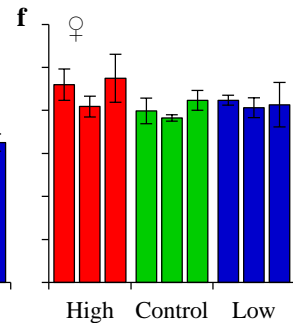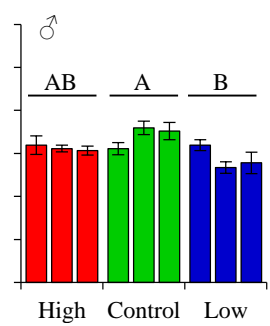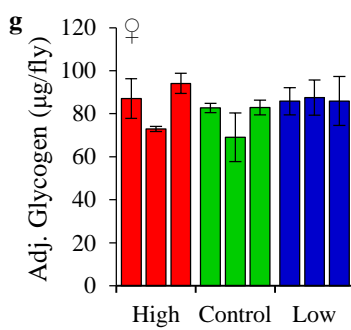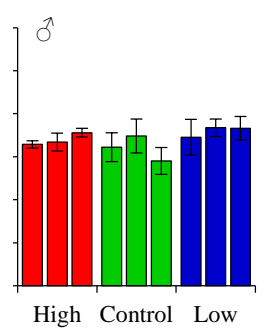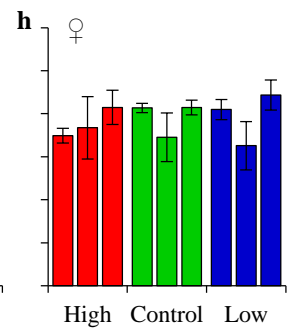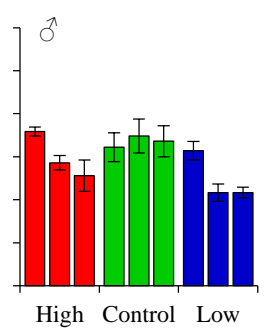

Supplement: Supplementary file 6 — Dry mass and measurements of metabolism. (a, b) Dry mass (N = 10), (c, d) adjusted triglyceride levels (N = 3), (e, f) adjusted glucose (N = 3), and (g, h) adjusted glycogen measurements (N = 3) for lines selected for increased and decreased behavioral responses to 4-ethylguaiacol and 4-methylphenol. Data shown are means ± SE for females and males (left and right columns, respectively). Letters indicate P < 0.05 using Tukey’s post hoc test. (PDF 207 kb) [file 12864_2017_4233_MOESM6_ESM.pdf]

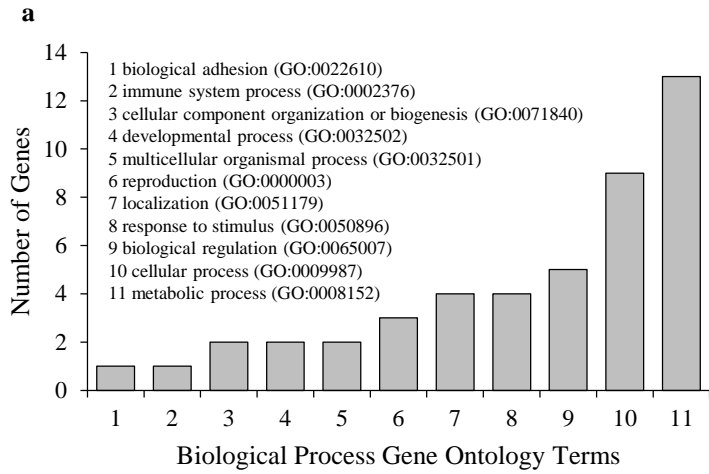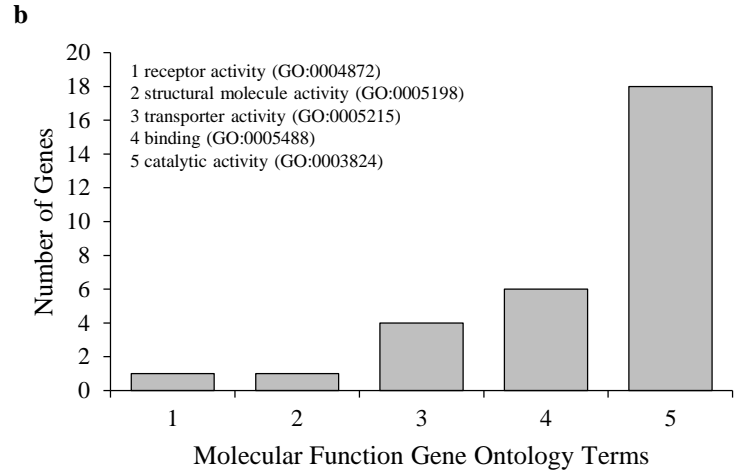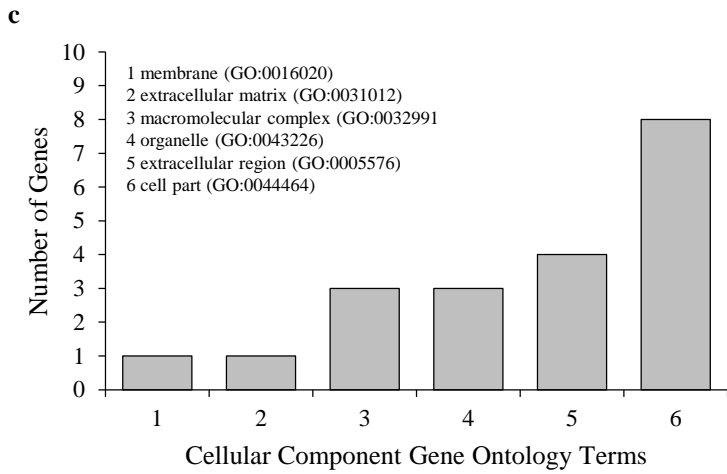

Supplement: Supplementary file 7 — Categorization of differentially expressed genes among lines selected for differences in behavioral responses to 4-ethylguaiacol into (a) biological process, (b) molecular function, and (c) cellular component gene ontology terms. (PDF 18 kb) [file 12864_2017_4233_MOESM7_ESM.pdf]
